# Multifunctional Carbon Nanotubes-Reinforced Surlyn Nanocomposites: A Study of Strain-Sensing and Self-Healing Capabilities

**DOI:** 10.3390/nano12162878

**Published:** 2022-08-21

**Authors:** Antonio del Bosque, Rocío Calderón-Villajos, María Sánchez, Alejandro Ureña

**Affiliations:** Materials Science and Engineering Area, Escuela Superior de Ciencias Experimentales y Tecnología, Universidad Rey Juan Carlos, C/Tulipán s/n, 28933 Madrid, Spain

**Keywords:** carbon nanotubes, structural health monitoring, self-healing, strain sensing, multifunctionality

## Abstract

Multifunctional nanocomposites based on carbon nanotubes (CNT)-reinforced Surlyn, which is a commercial ionomeric polymer, are manufactured by micro-compounding and hot-press processes. Multifunctionality is studied in terms of electromechanical response and self-healing abilities. The strain sensing analysis under tensile conditions shows ultra-high gauge factor (GF) values from 10 to 20 at low strain levels up to 10^6^ at high strain levels, and a decreasing sensitivity as CNT content increases because of the reduction in the tunneling distance between neighboring nanoparticles. The electromechanical response under consecutive tensile cycles demonstrated the robustness of the proposed materials due to the repeatability of both responses. With regard to mechanical properties, the addition of CNT induces a clear increase in Young’s modulus because the nanoparticles enable uniform load distributions. Moreover, self-healing capabilities are improved when 4 and 5 wt.% CNT are introduced because of the synergistic effect of the high thermal conductivity of CNT and their homogeneous distribution, promoting an increase in the thermal conductivity of bulk nanocomposites. Thus, by comparing the measured functionalities, 4 and 5 wt.% CNT-reinforced Surlyn nanocomposites showed a high potential for various applications due to their high degree of multifunctionality.

## 1. Introduction

Nowadays, there is an increasing interest in the development of multifunctional nanocomposites. This type of nanocomposites is characterized by fulfilling two or more different functionalities at the same time, typically, structural components with strain sensing capabilities [1,2], energy storage capacity [3,4,5], self-healing properties [6,7], etc.

In recent years, new technologies of Structural Health Monitoring (SHM) have received considerable interest because of their wide range of promising and unique properties. For this purpose, different sensing technologies are used to collect data that will be processed and interpreted to create a control system throughout the life cycle of an asset in terms of detecting, locating, and quantifying the damage that can occur [8,9,10]. Some conventional SHM techniques (acoustic emission, ultrasonic, Fiber Bragg, guided waves, etc.) are based on extremely complex mathematical tools and do not provide completely on-line information about the structure’s health, and, as a result, other options must be explored [11,12]. To solve this problem, polymeric nanocomposites doped with carbon nanoparticles such as carbon nanotubes (CNT), carbon black (CB), or graphene nanoplatelets (GNP) have emerged. The basis of this type of material lies in the fact that the addition of related conducting nanoparticles promotes the creation of conducting networks inside the polymer medium, which is electrically insulating. When above a critical volume fraction of conductive nanoparticles, called the percolation threshold, a drastic increment of several orders of magnitude of the electrical conductivity is promoted [13,14,15]. The percolation threshold depends on the characterized dimensions of conductive nanoparticles, ranging from 0.01–1 wt.% for CNT to 5–15% for GNPs, due to their 1D or 2D nature. Therefore, when damage or mechanical strain occurs, these electrical networks are affected, leading to significant changes in the electrical behavior of the nanocomposite. Furthermore, this type of nanocomposites should have high mechanical properties to act as a matrix in a fiber-reinforced composite or as a surface sensor on a structural component [16,17]. For this reason, GNP nanocomposites or flexible strain sensors are not suitable for these applications because of their poor mechanical behavior [18,19,20,21,22,23], despite the fact that new functionalities such as self-healing, self-adhesive, or high-performance communications have been achieved [24,25,26,27].

On the other hand, the investigation of strategies to develop self-healing functions for polymeric materials is an emerging area of research. Self-healing materials have the potential to significantly increase the working life and safety of structural components in a variety of applications. Several self-healing concepts for polymeric materials have been explored in the last 20 years, such as Diels–Alder reactions, ionomers, and supramolecular polymers. These mechanisms are based on the ability of some polymers to recross or link their chains. Typically, this mechanism needs an external stimulus to start, such as thermal, photonic, or chemical activation [7,28]. In this regard, ionomeric polymers usually comprise up to 20 mol.% of ionic species. The ionomer polymers are prepared in the form of chains with ionic groups and add a sufficient counterion to the polymer matrix. The properties of a self-healing ionomeric polymer can be altered by changing the ionic content. There are many different types of ionomers available, each with a varying ratio and type of cations used to neutralize acidic groups, such as sodium, magnesium, zinc, or lithium [29,30,31].

Therefore, this work is focused on the development of a multifunctional CNT-reinforced Surlyn, which is an ionomeric commercial polymer. In this regard, strain sensing and self-healing abilities have been explored. The electromechanical analysis allowed us to determine the mechanical properties and the sensitivity of the electrical response under applied strain, whereas the self-healing analysis indicated the influence of CNT on the volumetric recovery percentage. Moreover, a strain cycling test was carried out to demonstrate the repeatability of the electrical and mechanical responses and, thus, the robustness of the proposed nanocomposites. Finally, a summary of the results obtained shows a comparative study of the multifunctionality of the manufactured CNT-Surlyn nanocomposites to select the optimal CNT content.

## 2. Materials and Methods

### 2.1. Materials

Nanocomposites were manufactured with CNTs embedded in an ionomeric thermoplastic matrix. The ionomeric thermoplastic matrix used for this investigation was poly (ethylene-co-methacrylic acid) (EMAA) copolymer neutralized with 30 wt.% sodium, which is a polymer with self-healing capabilities under the commercial name of *Surlyn 8940^®^* (DuPont, Wilmington, DE, USA). Surlyn have a density of 95 g/cm^3^, a melt flow index of 2.8 g/10 min, and melting and freezing points measured by DSC of 94 °C and 63 °C, respectively. Multi-wall carbon nanotubes (MWCNTs) were produced via the Catalytic Chemical Vapor Deposition (CCVD) process, under the commercial name NC7000 (Nanocyl^®^, Sambreville, Belgium). They have an average diameter of 9.5 nm, a surface area of 250–300 m^2^/g, a length up to 1.5 μm, and a bulk density (EN DIN 60) of 66 kg/m^3^ with a 90% of carbon purity.

### 2.2. Manufacturing of CNT-Reinforced Surlyn Nanocomposites

Surlyn-based nanocomposites reinforced with CNTs were manufactured in two steps: first, CNTs were dispersed in Surlyn by the micro-compounding method; finally, their pellets were hot-pressed in a mold with the final shape of samples. CNT contents (4, 5, 6, and 7 wt.%) were selected to determine the degree of multifunctionality in terms of mechanical properties, electrical conductivity, and self-healing capability. 

On the one hand, CNTs were dispersed in Surlyn by the micro-compounding method using an Xplore MC 15HT machine (Barcelona, Spain). In the micro-compounder chamber, two counter-rotating conical screws were used to produce high shear forces on the mixture, promoting the breakage of CNT agglomerates. Moreover, the screws had small notches to induce certain reflux to favor CNT dispersion. Extruded filaments with a diameter of 3 mm were manufactured at 250 °C with a screw turning speed of 50 rpm. A study of the influence of compounding time on electrical conductivity measurements was carried out, taking filaments for each CNT content at 2, 4, 6, 8, and 10 min. In this regard, 10 min was selected as the optimal time to extract the filaments to manufacture the hot-pressed samples.

**Figure 1:**
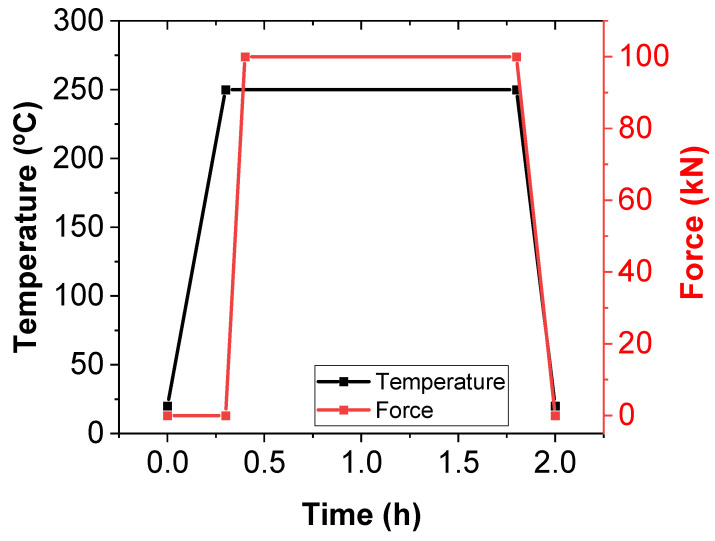
Hot-press force and temperature cycle for manufacturing bulk CNT–Surlyn nanocomposites from their pellets.

On the other hand, the extruded filaments were manually pelletized to a size of 3 mm in length, and they were placed in the metallic mold with the final shape of the specimens which was previously smeared with a layer of release agent based on polyvinyl alcohol (Castro Composites). Finally, they were hot-pressed in a Fortijne Presses LPB 300 machine (Delft, Netherlands), following the force/temperature cycle indicated in Figure 1.

### 2.3. Nanocomposite Characterization

#### 2.3.1. Microstructural Characterization

To evaluate the dispersion state reached for CNT–Surlyn nanocomposites with the micro-compounding method, the fracture surfaces under cryogenic conditions were analyzed by means of field-emission gun scanning electron microscopy (FEG-SEM), using an FEI TENEO machine (Thermo Fisher Scientific, Waltham, MA, USA). For proper characterization, the nanocomposite cryofractures were coated with 7 nm of gold.

#### 2.3.2. Electrical Conductivity Characterization

**Figure 2:**
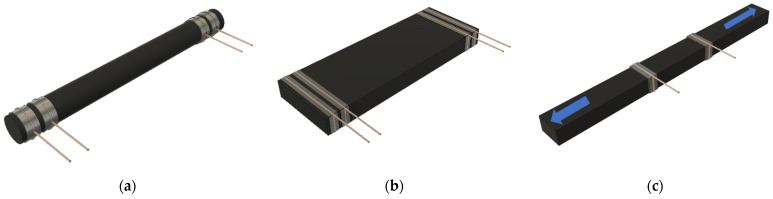
Specimens for electrical conductivity measurements in (**a**) extruded filaments and (**b**) hot-pressed samples, and (**c**) electromechanical tensile tests.

The electrical volume conductivity of extruded filaments and hot-pressed samples was measured using a Keithley 2410 Source Meter Unit (Cleveland, OH, USA). It was determined by calculating the slope of the current-voltage curve at 0–100 V for extruded filaments at low micro-compounding times, and at 0–10 V for extruded filaments at higher micro-compounding times and hot-pressed samples, because of the higher electrical conductivity expected in the last ones. Three specimens were evaluated for each condition, both extruded filaments (length of 50 mm and diameter of 3 mm) and hot-pressed (35 × 16 × 2 mm^3^). Four electrodes made of copper wires were attached with silver ink to the sample to make a four-probe measurement. Figure 2a,b show schemes of the placement of the electrodes in the extruded filament and hot-pressed specimens, respectively.

#### 2.3.3. Electromechanical Tests

Electromechanical properties of CNT–Surlyn nanocomposites were evaluated under tensile conditions using a ZWICK universal tensile machine (Ulm, Germany) equipped with a load cell of 5 kN. At least three tensile hot-pressed specimens with 60 × 8 × 6 mm^3^ dimensions of each CNT content were tested at a cross head speed of 2 mm/min and a preload of 25 N. 

The electrical resistance was measured simultaneously with the mechanical test using an Agilent DAQ970A (Santa Clara, CA, USA) data acquisition system with a DAQM902A module. Two copper wire electrodes were placed in the center and around the nanocomposite surface for this purpose, with 15 mm between electrical contacts. To reduce contact resistance, these electrodes were attached to the CNT–Surlyn nanocomposites with conductive silver ink, as shown in Figure 2c. Furthermore, samples were kept isolated from the testing machine by applying an adhesive layer to the grips.

The gauge factor (GF) of the proposed sensors is an essential factor for characterizing their electromechanical capabilities. It is a measure of electrical sensitivity to strain and can be defined as the ratio of the normalized electrical resistance ($\Delta R/R_{0}$) and strain (*ε*) induced in the material given by the Equation (1):

(1) $$\mathrm{GF}=\frac{\Delta R/R_{0}}{\varepsilon }$$

where ΔR is the electrical resistance increment and denominated $R_{0}$ is the initial electrical resistance.

Moreover, to study the repeatability of the electromechanical response of the developed nanocomposites, a tensile specimen of each CNT content was subjected to tensile cycling for 200 cycles and up to 1% strain level, at a fixed rate of 20 mm/min.

#### 2.3.4. Self-Healing Tests

The self-healing capabilities of nanocomposites were evaluated by comparing indentation damage before and after the self-healing. Three surface indentation damages were applied by a constant load of 10 kg for 15 s using the Shore D ASTM D2240-05 standard. For this purpose, the volumetric recovery percentage, V, was calculated as indicated in Equation (2):

(2) $$V\;\left(\%\right)=\frac{V_{0}-V_{f}}{V_{0}}\cdot 100$$

where $V_{0}$ is the initial volume of the indentation damage and $V_{f}$ is the final volume after the self-healing process that consisted of heating the sample with the damage indentation in an oven at 80 °C for 1 h. The temperature and duration of heating were chosen based on previous investigations [32]. The different volumes were measured using a Zeta-20 Instruments 3D optical profilometer (Milpitas, CA, USA) which generated 3D micrographs. These micrographs were obtained before and after the self-healing process and subsequently processed with the Mountain Map Premium 7.1 software (Besancon, France).

## 3. Results and Discussion

The degree of multifunctionality of CNT-Surlyn nanocomposites were analyzed, in terms of electrical, electromechanical, and self-healing capabilities. For this purpose, it is very important to understand the role and the dispersion of CNT in Surlyn polymer matrix.

### 3.1. Electrical Conductivity Measurements

**Figure 3:**
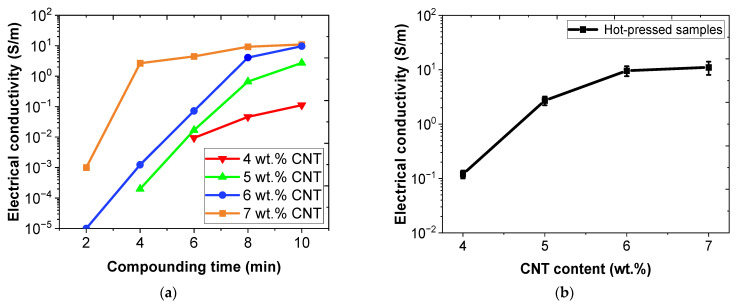
Electrical conductivity measurements of (**a**) extruded filaments at different compounding times and (**b**) hot-pressed samples as function of CNT content.

Figure 3 shows the values of electrical conductivity measurements for the different CNT contents tested for extruded filaments at different compounding times and for hot-pressed samples. The effect of both CNT content and compounding time can be analyzed.

First, as expected, it can be observed that an increasing amount of CNTs rebounded into a higher electrical conductivity due to the creation of more electrical pathways within the material, reaching values of near 10 S/m to 6 and 7 wt.% CNT. These values are higher than those reported with a similar dispersion procedure and CNT content in thermoplastic matrixes [33,34]. Furthermore, the electrical conductivity results proved that the percolation threshold, that is, the minimum CNT fraction that allows the creation of electrical pathways, was less than 4 wt.%. In this regard, it is important to note that samples with 3 wt.% CNT were electrically unconducive with this processing.

Secondly, the higher the compounding time, the higher the electrical conductivity values, as Figure 3a shows. This is because, in the micro-compounder chamber, two counter-rotating conical screws were used to produce high shear forces on the mixture, promoting the breakage of CNT agglomerates by rupture and erosion mechanism and, thus, the higher electrical conductivity [35]. Moreover, nanocomposites needed a longer compounding time to create effective electrical pathways when it comes to samples with a lower concentration of CNT, as expected. For example, 7 wt.% of extruded filaments were electrically conductive at 2 min in the chamber, whereas 4 wt.% started to show electrical conductivity at 6 min. Here, 10 min was selected as the optimum compounding time because the electrical conductivity values started to stabilize because the high shear forces produced on the mixture also induced breakage of the CNTs themselves [36].

On the other hand, similar electrical conductivity was observed when comparing the extruded filaments (Figure 3a) with the hot-pressed samples (Figure 3b) for the same CNT contents, which means that there were no important changes in the CNT distribution during the hot-press process. 

**Figure 4:**
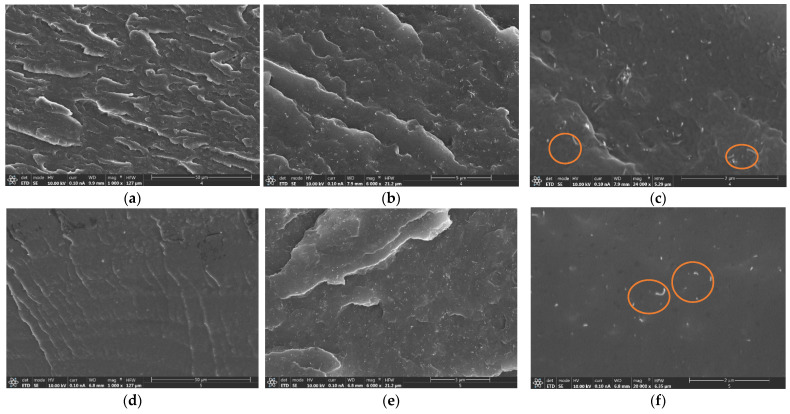

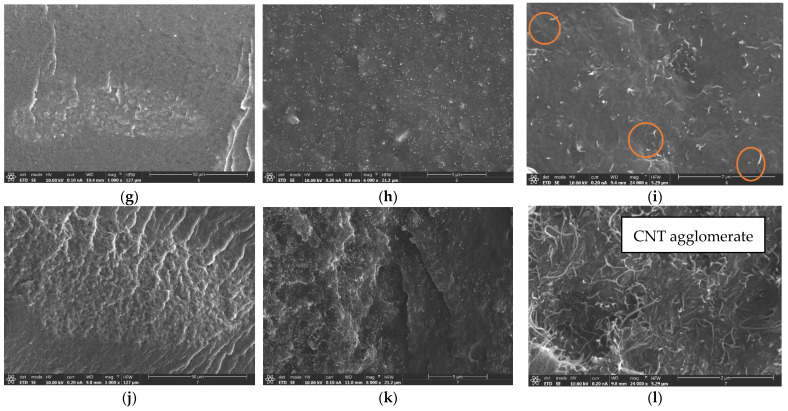
FEG-SEM images of the CNT–Surlyn nanocomposites at (**a**–**c**) 4, (**d**–**f**) 5, (**g**–**i**) 6, and (**j**–**l**) 7 wt.% CNT. The left, center, and right images are at 1000, 6000, and 24,000 magnifications, respectively.

The good CNT dispersion of most of the samples was also confirmed by the FEG-SEM analysis of cryofracture surfaces, as shown in Figure 4. Nanocomposites with lower contents (4 and 5 wt.% shown in Figure 4a–f) promote a very homogeneous distribution, with the absence of larger aggregates. However, nanocomposites with higher contents (6 and 7 wt.% shown in Figure 4g–l) present some larger aggregates, as clearly shown in Figure 4l. The right images showed a high-magnification detail of CNT-Surlyn nanocomposites, with some individual CNT marked with orange circles. Despite the high CNT content (4–7 wt.%), a homogeneous distribution of nanoparticles in the samples was reported.

### 3.2. Electromechanical Tests

**Figure 5:**
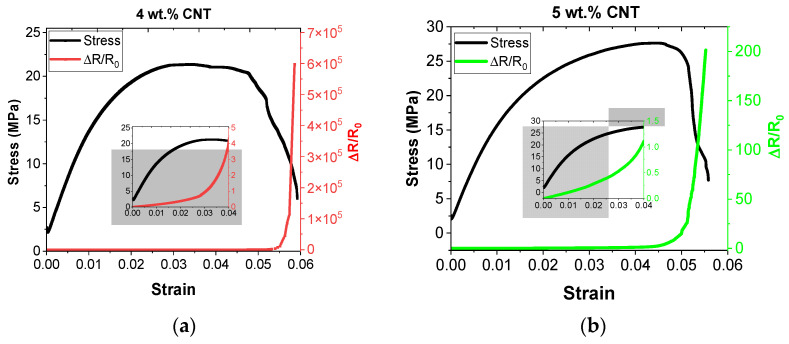

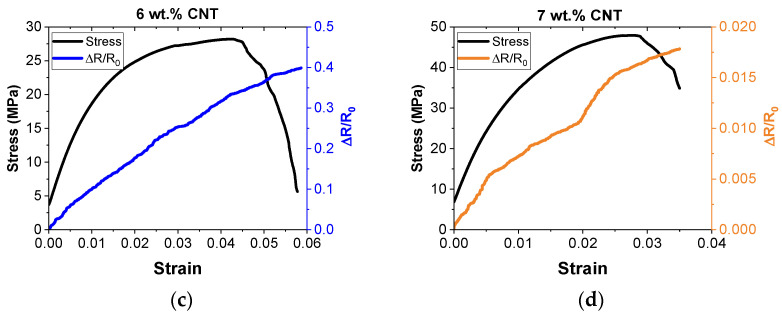
Electromechanical response of CNT–Surlyn nanocomposites under tensile conditions for (**a**) 4, (**b**) 5, (**c**) 6, and (**d**) 7 wt.% CNT.

Therefore, after analyzing the electrical properties of the CNT–Surlyn nanocomposites, the electromechanical response was studied in detail. In this context, Figure 5 summarizes some representative curves of the electromechanical behavior of the nanocomposites under tensile load.

When the electromechanical curves in Figure 5 were examined, the electrical resistance exhibits a linear–exponential behavior with applied strain. This fact has been widely reported [21,23], and it is related to the prevalence of tunneling mechanisms in the electrical network formed in nanocomposites, in which migration of electrons between nanoparticles takes place when there are two adjacent nanoparticles with a distance lower than 2 nm inside an insulator media. According to Simmons [37], the electrical resistance associated with the tunneling mechanism ($R_{tunnel}$) follows an exponential behavior with the tunneling distance (*t*), that is the distance between adjacent nanoparticles, as shown in Equation (3).

(3) $$R_{tunnel}=\frac{h^{2}t}{Ae^{2}\sqrt{2m\lambda }}exp\left(\frac{4\pi t}{h}\sqrt{2m\lambda }\right)$$

where *h* is Planck’s constant, *m* and *e* are the electron mass and charge, *A* the cross-sectional area of CNT nanoparticles, and *λ* the height barrier of the matrix. 

**Figure 6:**
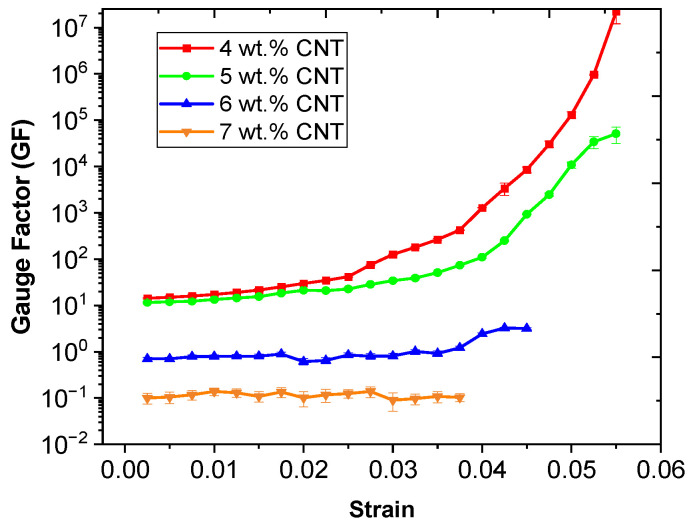
Gauge factor value of CNT–Surlyn nanocomposites as function of applied strain.

In this regard, a pronounced exponential electrical response with the applied strain can be observed for lower CNT contents (4 and 5 wt.%) in comparison with a more linear response of higher CNT contents (6 and 7 wt.%), attributed to the prevalence of the tunneling mechanism in detriment of the contact mechanism between nanoparticles [38]. The gauge factor (GF) of the reported nanocomposites can be calculated using these electromechanical curves, which is a typical measure of electrical sensitivity to strain. Figure 6 denotes the GF obtained as a function of CNT content and applied strain. 

It can be observed that the GF increases with decreasing CNT content, as expected, because the closer to the percolation threshold, the higher the sensitivity of the system [39]. Here, by increasing the CNT content (the number of nanoparticles), the average distance between nanoparticles decreases and, therefore, the exponential variation in the electrical resistance due to tunneling mechanisms is less prevalent, leading to lower electrical sensitivities, that is, lower GF. In this regard, 4 and 5 wt.% nanocomposites show an ultrasensitive behavior, reaching a GF of 10–12 at *ε* = 0.01 and 10^4^–10^6^ at *ε* = 0.05, which are much higher than those found in conventional metallic gauges for strain sensing purposes, which is around 2. On the other hand, 6 and 7 wt.% nanocomposites have a much lower GF with the applied strain, between 0.2 and 2.

**Figure 7:**
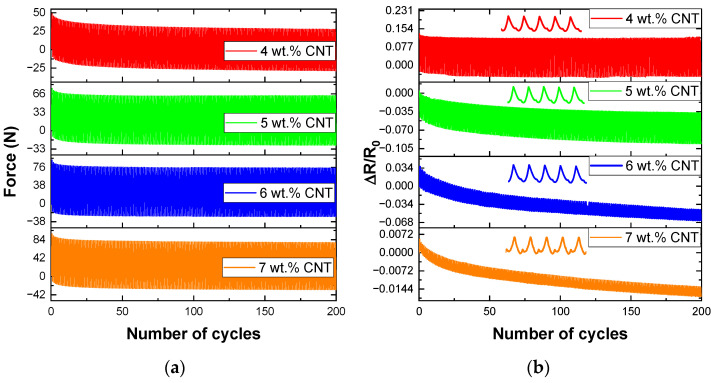
Cycling response of nanocomposites at 1% strain levels. (**a**) mechanical and (**b**) electrical response under 300 cycles. A detail of 150–155 cycles is shown in electrical response.

Furthermore, Figure 7 shows the mechanical and electrical response of nanocomposites under tensile cycling load at 1% strain levels. The goal is to demonstrate the repeatability of the electrical and mechanical response and, as a result, the robustness of the proposed nanocomposites. To prove their potential to detect low damage and strain, they were tested at a cycling strain up to 1% strain level. Here, two significant facts can be stated: on the one hand, the response under cyclic load is in agreement with the quasi-static electric response previously reported and, the resistance changes decrease with increasing CNT content, as expected; on the other hand, the sensitivity of the reported sensors is constant over the 200 cycles because the change in the electrical resistance remains nearly constant (distance between the peak and baseline) for each consecutive cycle. It denotes that the electrical network is fully recovered after applying the load, with the absence of microcracks. Moreover, although the sensitivity is maintained throughout the cycles, the electrical curves shown in Figure 7b followed an initial downward trend that then tended to stabilize, indicating that the resistance tends to decrease with the number of cycles. This is because of the emergence of new conductive networks and their equilibrium state after a period of adjustment, which is more prevalent with the higher CNT content, as has been widely reported [40]. Therefore, the use of CNT–Surlyn nanocomposites for Structural Health Monitoring purposes has been demonstrated since they can detect a robust electromechanical response at low strains level.

In this type of nanocomposites, it is important to analyze the influence of CNT on mechanical properties. Table 1 and Figure 5 show the values of mechanical properties and stress–strain curves of nanocomposites, respectively. Here, the addition of CNT to the Surlyn matrix causes a clear increase in Young’s modulus, a slight increase in tensile strength, and a decrease in strain at failure. First, these results revealed no degradation of the Surlyn polymer because of the double thermal procedure, i.e., filament extrusion and hot-press procedure. The improvement of Young’s modulus and tensile strength can be explained by the homogeneous CNT dispersion in 4 and 5 wt.% shown in Figure 4a–f, which enables uniform load distributions and, thus, reduces the load concentration [41,42]. However, a slight reduction in strain at failure was observed with the higher CNT content, which is accentuated in the 7 wt.% sample because it showed more CNT agglomerates (Figure 4g–l). These agglomerates promote the generation of free space between the matrix and the reinforcement acting as a stress concentrator with the applied strain. This tendency is in agreement with similarly reinforced nanocomposites [2,16,39].

### 3.3. Self-Healing Test

**Figure 8:**
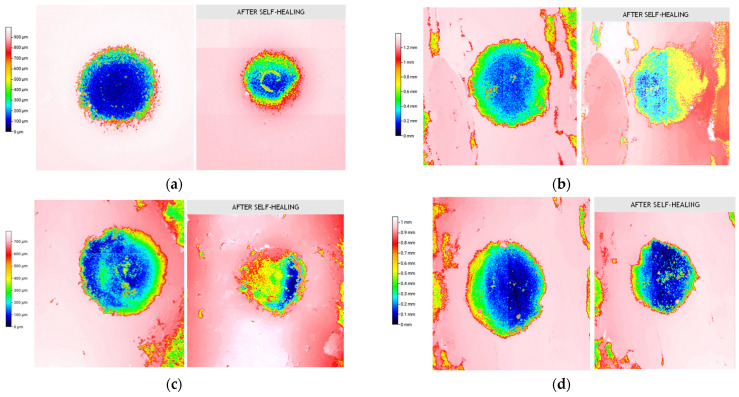

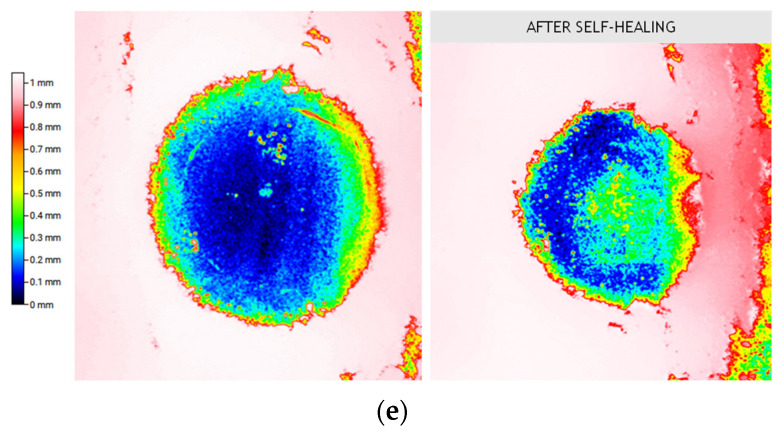
Profilometer micrographs of the (**a**) neat, and reinforced Surlyn with (**b**) 4, (**c**) 5, (**d**) 6, and (**e**) 7 wt.% CNT. The left and right images show the sample before and after self-healing, respectively.

Figure 8 shows profilometer micrographs of the neat and reinforced Surlyn before and after self-healing activated by convection heat in an oven at 80 °C for 1 h. Moreover, Table 1 indicates the volumetric percentage of damage recovered of the samples. 

Here, it was corroborated that the volumetric recovery calculated for neat Surlyn (66 ± 2%) coincided with that reported by the commercial manufacturer (68 ± 5%). In this regard, the incorporation of CNT to the Surlyn matrix causes two different effects: for low CNT contents (4 and 5 wt.%) a notable increase in self-healing was observed, reaching values of up to 80 %; however, for high CNT contents (6 and 7 wt.%) a decrease in the recovered volume was reported, even losing self-healing capacity with respect to pure Surlyn.

On the one hand, the improvement in the self-healing values can be explained by the synergistic effect of the high thermal conductivity of the CNTs (around 3000 W/mK) and their homogeneous distribution, as reported in Figure 4a–f, promoting an increase in the thermal conductivity of bulk nanocomposites and, therefore, in the volumetric recovery percentage [43,44]. On the other hand, the decrease in the self-healing values is because the increase in the number of nanoparticles and their tendency to agglomerate (as indicated in the Figure 4g–l) can prevent the “ion hopping” and the elastic movement of the polymeric chains, which are responsible for the self-healing process [6,24,42].

### 3.4. Analysis of Optimum Conditions for Application

The selection of an optimum CNT–Surlyn nanocomposite depends on its multifunctionality. The multifunctionality is studied in terms of electrical conductivity, electrical sensitivity (Gauge Factor), mechanical properties (Young’s modulus), and self-healing properties (volumetric percentage of damage recovered). Here, a radar chart was constructed to obtain a complete overview [45]. For this analysis, neat Surlyn is not considered, because it is not multifunctional.

In this radar chart, each measured property has been rescaled from 0 to 1, with 1 denoting the best performance for that property. As a result, the “best” material will have a factor of 1, whereas the rest of the conditions were rescaled based on the value of this property. This reescalation follows a linear trend for the gauge factor, Young’s modulus, and self-healing properties. However, because of the high sensitivity to small variations in electrical conductivity, it has been rescaled using a logarithmic trend, with 1 symbolizing the highest measured electrical conductivity, and 0 denoting the conductivity at the percolation threshold, which has been fixed at 10^−5^ S/m as observed in this work.

**Figure 9:**
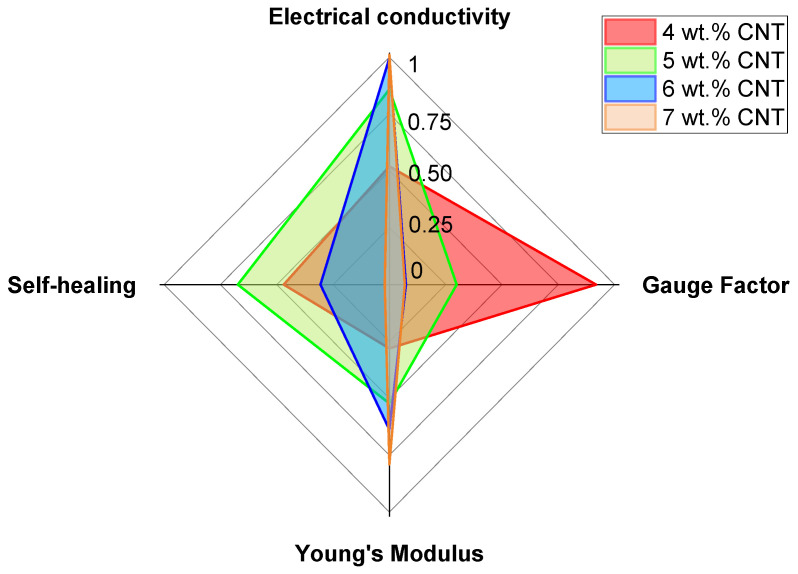
Radar chart of the different CNT–Surlyn nanocomposites to compare their multifunctionality, scaled from 0 to 1.

Figure 9 shows the calculated values of the mentioned properties. Here, the area occupied by the curves gives an idea of the multifunctionality of nanocomposites. In this regard, the nanocomposites with 4 and 5 wt.% CNT show higher multifunctionality than those containing 6 and 7 wt.% CNT. Surlyn reinforced with 4 and 5 wt.% CNT seems to be a very promising solution for accomplishing all the analyzed functionalities, due to the good balance conferred by a good CNT dispersion. Furthermore, depending on whether electrical sensitivity or self-healing capability is prioritized, nanocomposites with 4 or 5 wt.% CNT can be selected for each final application, respectively. In any case, the CNT–Surlyn nanocomposites showed a high potential for various applications due to their high degree of multifunctionality.

## 4. Conclusions

The electromechanical response and self-healing abilities of multifunctional nanocomposites made of CNT-reinforced Surlyn were investigated. The analysis of electrical conductivity values showed that the percolation threshold was less than 4 wt.% CNT and the extruded filaments reached similar conductivity to hot-pressed samples for the same CNT content. The strain monitoring response under tensile conditions showed that an increase in the CNT content leads to a decrease in the sensitivity because of the reduction in the tunneling distance between neighboring nanoparticles, reaching a GF for 4 and 5 wt.% CNT of 10–12 at *ε* = 0.01 and 10^4^–10^6^ at *ε* = 0.05, which are much higher than those found in conventional metallic gauges for strain sensing purposes. Moreover, the electromechanical response under tensile cycles demonstrated the robustness of nanocomposites. The addition of CNT to the Surlyn matrix causes a clear increase in Young’s modulus and a slight increase in tensile strength, explained by the good CNT distribution which enables uniform load distributions. The improvement in the self-healing values when 4 and 5 wt.% CNT are introduced can be explained by an increase in the thermal conductivity of bulk nanocomposites. Therefore, by comparing the measured properties, Surlyn reinforced with 4 and 5 wt.% CNT seems to be a high potential solution for accomplishing all the analyzed functionalities, selecting one content or another depending on which properties are most critical in the final application.

## Figures and Tables

**Table 1:** Values of the mechanical and self-healing properties for neat Surlyn and CNT–Surlyn nanocomposites.

	Neat	4 wt.% CNT	5 wt.% CNT	6 wt.% CNT	7 wt.% CNT
**E (GPa)**	1.12 ± 0.07	1.22 ± 0.14	1.41 ± 0.16	1.50 ± 0.09	1.62 ± 0.20
**σ_TS_ (MPa)**	23.05 ± 2.70	21.37 ± 3.55	27.64 ± 4.45	28.09 ± 3.12	28.35 ± 4.98
**Ɛ** **_f_**	0.061 ± 0.005	0.059 ± 0.003	0.056 ± 0.007	0.058 ± 0.010	0.035 ± 0.009
**V (%)**	66 ± 2	73 ± 5	83 ± 2	65 ± 5	51 ± 11

E: Young’s modulus; σ_TS_: tensile strength; Ɛ_f_: strain at failure; V: volumetric percentage of damage recovered.

## Data Availability

The data presented in this study are available on request from the corresponding authors.
